# Potential impacts of nanoparticles integration on micropropagation efficiency: current achievements and prospects

**DOI:** 10.3389/fpls.2025.1629548

**Published:** 2025-10-07

**Authors:** Gurudayal Ram Guru, Pramod W. Ramteke, Csilla Veres, Csaba Vágvölgyi

**Affiliations:** ^1^ Centre for Tissue Culture Technology, Jacob Institute of Biotechnology and Bioengineering, Sam Higginbottom University of Agriculture, Technology and Sciences, Prayagraj, Uttar Pradesh, India; ^2^ Department of Molecular Biology and Genetic Engineering, Rashtrasant Tukadoji Maharaj Nagpur University, Nagpur, Maharashtra, India; ^3^ Department of Biotechnology and Microbiology, Faculty of Science and Informatics, University of Szeged, Szeged, Hungary

**Keywords:** nanoparticles, micropropagation, nanotechnology, acclimatization, green synthesis, nanotoxicity, regulatory framework

## Abstract

The use of nanoparticles (NPs) in plant tissue culture systems represents a new approach to improve the efficiency of micropropagation. Owing to their nanoscale size, high surface area concomitant with volume, and controllable and targeted release, researchers have tested the experimental benefits of NPs in various ways during each phase of *in vitro* propagation, which include enhancing surface sterilization to reduce microbial contamination, the targeted uptake of specific macro-and micronutrients, regulating plant hormonal activity to enhance callogenesis, increased shoot multiplication and rooting, and increasing the survival rate during acclimatization. In addition, some situations where NPs are applied can reduce oxidative stress and regulate hormonal pathways, which will stabilize the physiological state of the plant and support better developmental integrity of the regenerating plantlets. In moving forward with the application of nanoparticles, the major limiting factors are nanotoxicity, persistence in the environment, species specificity, and the lack of an established regulatory framework. In this review, the recent published successes in NP-mediated micropropagation are summarized, how they impart their effects in plant science at the cellular and molecular levels are explained, and potential future innovations such as green-synthesized nanomaterials and new smart delivery platforms are also identified. Realizing the full potential of nanotechnologies for application with micropropagation will be critical for developing scalable, sustainable, and precision agricultural production systems.

## 1 Introduction

The process of plant micropropagation has quickly become an essential technology in the fields of agriculture, horticulture, forestry, and conservation biology since it allows for rapid multiplication of genetically identical and pathogen-free high-throughput plant material without the risk of contamination (Engelmann and Dussert, 2000 ; Cassells, 2002 ; El-Esawi, 2016). Plant micropropagation is a tissue culture process *in vitro* and is broadly defined as the process of obtaining plant material from plant tissues under controlled laboratory conditions. In its brief evolution from concept to application, micropropagation has advanced significantly over the past several decades in various aspects of media optimization, phytohormone use, and culture systems (Gupta et al., 2020a). However, hurdles still exist in micropropagation, such as microbial contamination, nutrient use inefficiencies, hormonal imbalances, oxidative stress, and suboptimal processes for acclimatization (Abdalla et al., 2022). In response to these issues, the continued development of nanotechnology, particularly the use of nanoparticles, signifies a breakthrough opportunity to overcome some of the challenges in micropropagation and increase its efficiency (Bhandari et al., 2022).

As a new field for creating new materials that exploit manipulation of matter at the nanoscale of 1–100 nanometers, nanotechnology has exciting potential applications in the life sciences as a whole (Tawade and Wasewar, 2023; Singh, 2023; Singh et al., 2024a, Singh et al., 2024b). In plant biotechnology, exciting investigations using NPs are beginning to demonstrate their highly unique physicochemical properties, such as an increased ratio of surface area to volume, enhanced reactivity and surface chemistry, which can be easily tuned and modified, with controlled release and targeted delivery abilities. These properties put NPs in an excellent position for multiple purposes in micropropagation, such as (but not limited to) antimicrobial sterilization, cation and hormone delivery, activating or steering morphogenic pathways, changing plant responses to oxidative stress, and improving plant metabolism, especially in sensitive growth and development phases (Newsom, 2022).

One of the first uses, and perhaps one of the most researched uses, of nanoparticles in micropropagation is their antimicrobial properties (Karimi, 2019). Standard tissue culture systems are often highly vulnerable to bacterial and fungal contamination, arising from either endogenous or exogenous sources and ultimately resulting in a meaningful loss of significant germplasm (Dangariya et al., 2020). Numerous metallic nanoparticles, such as silver (AgNPs), zinc oxide (ZnO NPs), copper oxide (CuO NPs), and titanium dioxide (TiO_2_ NPs), have ambiguous but strong, broad-spectrum antimicrobial effects on a variety of plant pathogens (Mandal and Sahu, 2021). There are multiple potential mechanisms by which pathogen mechanisms can interfere; these include altering microbial membranes, generating reactive oxygen species (ROS), and disrupting function at the levels of reaction and genetics (Mostafa et al., 2018). The use of nanoparticles offers a high probability of reducing or even eliminating toxic chemical sterilants that pose potential hazards to explant viability and environmental health (Tariq et al., 2020).

NPs may have the ability to serve as smart carriers of nutrients and phytohormones (López-Valdez et al., 2018) due to the challenges of precipitation, slow nutrient uptake, and hormone degradation present in tissue culture. Nanofertilizers and nanoelicitors may have potential to enable the precision of specific control of plant growth regulators by stage, reducing variability and simultaneously improving the reliability and reproducibility of plant tissue culture (Javed et al., 2022; Nagargade et al, 2022).

Notably, nanoparticles may also be useful for addressing oxidative stress, an intrinsic negative side effect *in vitro* that affects cell viability and morphogenic responses. Some nanoparticles, such as ZnO, TiO_2_, and SiNPs, activate antioxidant defense systems by increasing the levels of antioxidants, such as superoxide dismutase (SOD), catalase (CAT), and peroxidase (POD). They help maintain the redox balance of the cells, ultimately reducing physiological stress, the amount of tissue browning, the survival of explants, and the ability to support plant regeneration. Additionally, these nanoparticles interact directly with plant signal transduction networks, leading to the upregulation or downregulation of gene expression and thus creating biocompatible devices for lucrative and ultimately establishing vigorous plant growth and resilience during exposure to *in vitro* conditions (Jalil and Ansari, 2021).

Owing to the promising nature of these findings, the incorporation of nanoparticles in micropropagation is fraught with significant scientific, economic, and regulatory issues. Perhaps the most important issue is nanoparticle toxicity, which can be distinguished in three areas, i.e., cytotoxicity, genotoxicity, or developmental abnormality, depending on the type, concentration, and time of exposure to the nanoparticles. High levels of Ag and CuO nanoparticles lead to oxidative damage, membrane disruption, and other specific types of inhibition of cell division. Second, because there is no protocol for the synthesis, characterization, and use of nanoparticles, it is difficult to replicate and compare them to one another since the variability hinders any efforts to establish best practices and therefore limits the expansion or scale-up of nanoparticle-assisted micropropagation systems (Athar et al., 2022).

Environmental considerations regarding potential nanoparticle bioaccumulation, leaching into soils and water systems, and probable impacts on non-target organisms increase the need for comprehensive risk assessment. Current research addresses the fate of nanoparticles after they have been explanted, what the nanoparticles are doing with the beneficial soil microbiota, and whether any of these materials are entering the food chain after propagation. Therefore, there are significant regulatory gaps associated with the use of engineered nanomaterials in agriculture (including tissue culture). There are limited regulatory frameworks associated with safety assessment, labeling, and disposal, thus, there is considerable ambiguity with respect to commercial adoption and large-scale implementation (Singh and Gurjar, 2022).

In addition, the economic feasibility of using nanoparticles in micropropagation also plays an important role. There are efficacy data for micropropagation using microgram-level doses in laboratory-scale studies, but for propagation on an industrial scale, bioprofessionals will require grams to kilograms of the materials for production efficiency, leading to questions of cost-effectiveness and decision-making related to logistics and quality control. In addition to the synthesized engineered nanoparticles, it is possible to use green synthesis methods with plant extracts or microbial systems to generate nanoparticles of lower environmental concern and possibly lower economic cost, but these methods require optimization to yield the same level of reproducibility and functional capacity that has been defined from chemically synthesized nanoparticles (Khanna et al., 2023).

Taking all these matters into account, the scope of this review is to address the current knowledge of the use of nanoparticles in micropropagation by discussing their mechanistic role, advantages, and challenges. Recent advances in the forms of nanoparticle-mediated antimicrobial agents, nutrient- or hormone-expressing particles, redox regulation, and metabolic pathway modulation are emphasized. However, we also look to address the issues of dose versus response, safety, environmental concerns, regulations, and costs associated with the *in vitro* use of nanoparticles. In addition to the features of nanoparticles currently found in the literature, we also dissect the future opportunities of biodegradable and smart nanoparticles and the integration of nanoparticle application with precision agriculture and ‘omics’ science along with the greater impact on sustainable plant biotechnology.

By offering a systematic examination of both the advantages and the limitations of integrating nanoparticles into micropropagation, we seek to provide a foundational understanding for researchers, technologists, and policy-makers. Ultimately, in light of rapidly advancing knowledge in nanotechnology, we seek to build a roadmap for rational and safe design and scalable application of innovative solutions to improve the efficiency, reliability, and sustainability of plant micropropagation systems in the 21st century.

## 2 Effects of nanoparticles on micropropagation efficiency

**Figure 1:**
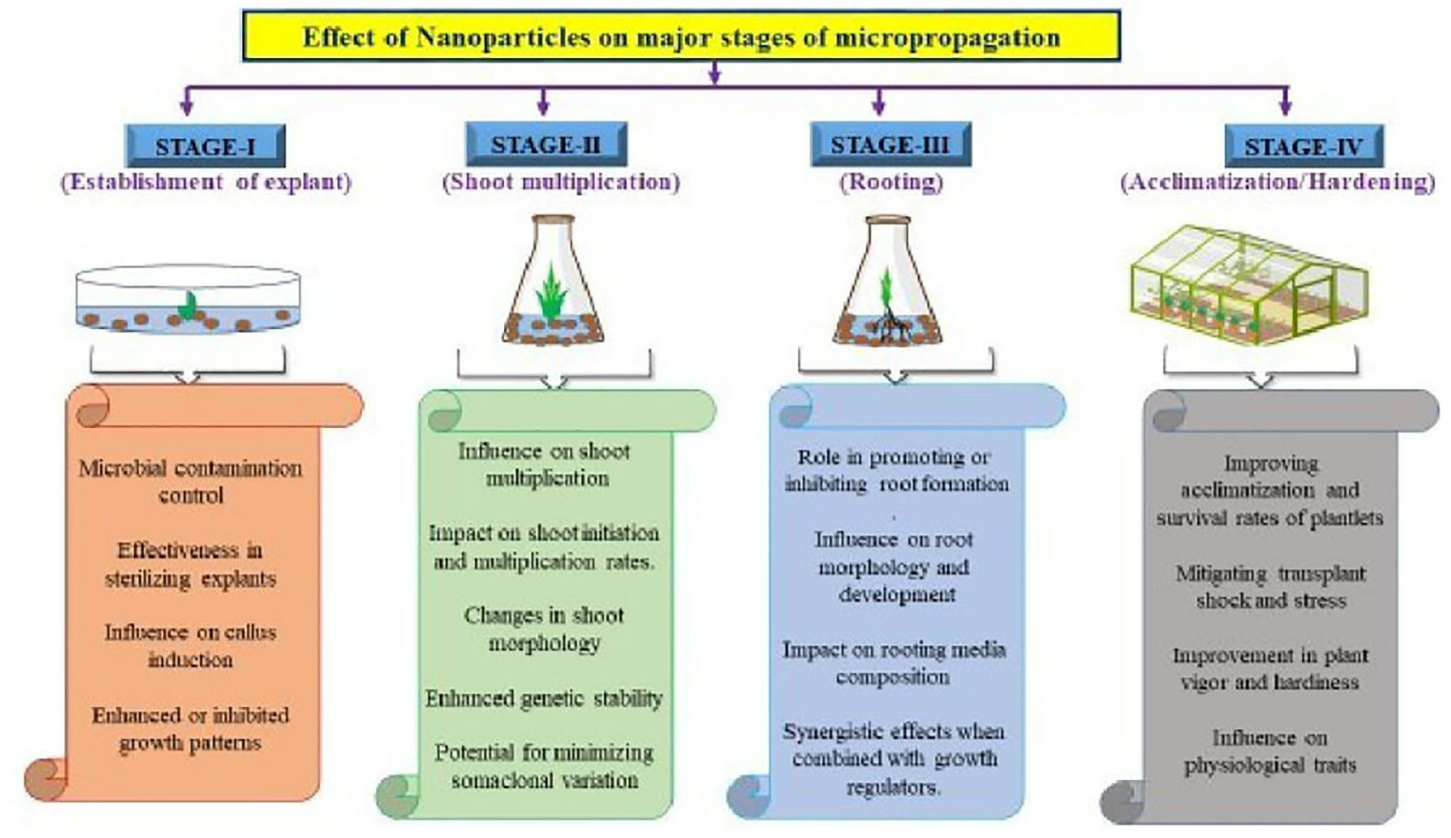
Effect of nanoparticles on major stages of micropagantion.

Recent advancements in the field of science associated with nanotechnology offer a relatively new technology to ameliorate some of the issues in *in vitro* plant culture. The physicochemical properties of nanoparticles enable them to be highly useful in enhancing the stimulation of micropropagation at all major stages (**Figure 1**). Various investigations have been conducted in recent years on important crops to assess the effects of nanoparticles at various major stages of micropropagation, as discussed below:

### 2.1 Initiation/establishment stage

The initiation stage of micropropagation could be enhanced by the application of nanoparticles such as silver and copper oxide nanoparticles, which, as a consequence, not only improve explant viability but also reduce contamination and enhance morphogenic potential to develop successful plant tissue culture (**Table 1**). Microbial contamination is widely perceived as the main bottleneck of plant tissue culture, which limits the efficacy of the micropropagation method. Common sterilization practices and techniques used in plant tissue culture can also damage plant tissues and/or produce toxic waste when not performed properly through the use of sodium hypochlorite, ethanol, or mercury chloride. However, nanoparticles, particularly silver nanoparticles, have gained attention as eco-friendly and effective alternatives for sterilization in tissue culture (Salisu et al., 2014).

**Table 1:** Effect of nanoparticles on initiation/establishment stage of micropropagation.

S. no.	Plant species	Explant	Type of nanoparticles	Average size of NPs	Synthesis method	Validation and characterization	Optimal concentration range	Effective concentration for contamination control	Mechanism of action	Effect on regeneration efficiency	Limitations observed	References
1.	Olive (*Olea europaea* cv. Sourani)	Nodal cuttings	AgNPs	6.68 nm to 15 nm.	Chemical reduction method	UV/VIS Spectrophotometer and TEM	0, 100, 200, and 400 mg L	400 mg/L	Interacts with bacterial cell membrane proteins, causing cell death. Release silver nanoparticles as disinfectants, destroying bacteria, fungi, and viruses.	Highest bud sprouting percentage at 10 mg/L Highest shoot length at 20 mg/L	-----	Hegazi et al. (2023)
2.	Lemon grass (*Cymbopogon citratus*).	Nodal Segment	AgNPs	----------	Biological method (Green synthesis)	UV/VIS Spectrophotometer and TEM	10 to 40 ml/L	40 ml/L	Disrupt cell membrane structure, leading to cell death . Deplete intracellular ATP, collapsing plasma membrane potential	Reduced bacterial contaminants	Total elimination of fungal contamination was not achieved with AgNPs	Salisu et al. (2014)
3.	Rose (*Rosa hybrida*)	Nodal Segment	AgNPs	---------	---------	---------------	0, 50, 100 and 150 ml/L	100 mg/L	Silver ions interact with proteins and DNA, inhibiting respiratory processes Inhibition of cell division and damage to bacterial cell envelopes occur.	Reduced bacterial contamination and phenolic exudation rate.	High concentrations of Nano-Silver slow down explant regeneration and may destroy explants and was ineffective against fungal contamination	Shokri et al. (2014)
4.	Date palm (*Phoenix dactylifera* L.) cv. Barhee.	Shoot tip	AgNPs	---------	------------	---------------	1,5,10,20 mg/L	5 mg/L	Silver nanoparticles release low levels of silver ions, combating microorganisms	Enhanced survival of explants (88.89%)	Higher concentrations of silver nanoparticles caused the highest explant mortality	El-Sharabasy et al. (2017)
5.	Valerian (*Valeriana officinalis* L.)	Nodal segment	AgNPs	35 nm	Chemical method	TEM	25, 50, and 100 mg/L	100 mg/L	Silver ions interact with -SH groups and DNA bases, inhibiting processes	Good potential for removing the bacterial contaminants	High exposure times caused explants to bleach, affecting results	Abdi et al. (2008)
6.	*Arabidopsis thaliana* and *Brassica napus*	Seed	AgNPs	-----------	Chemical method	--------------	40-80 mg/L	80 mg/L	Silver nanoparticles destabilize plasma membrane potential, leading to cell death .	Improved plant growth and the reduction of fungal and bacterial contamination	High concentrations (500 and 1000 mg/L) inhibited plant growth	Soltanloo et al. (2010)
7.	*Arabidopsis thaliana* cv. Col-0 and Tomato (*Lycopersicon* *esculentum* cv. Micro-Tom)	Seeds	AgNPs	-----------	Chemical method	---------------	100 mg/L	100 mg/L	Silver nanoparticles interfere with electron transport and bind to DNA . They interact with cell membranes, causing proton leakage and ion discharge	Can be used as a disinfectant for plant seeds, especially for *in vitro* cultures Concentration of 150 mg/L showed toxicity for rapeseed seeds	Higher concentrations (1000-2000 mg/L) resulted in no seed germination	Mahna et al. (2013)
8.	GxN15 (hybrid of almond · peach) rootstock	Nodal Segments	AgNPs	--------------	--------------	--------------	100 and 150 mg/L	100 and 150 mg/L	Nano-silver binds to microbial DNA, preventing bacterial replication Ag ions destroy cell membranes of microorganisms	Reduced internal and external contaminations	High concentration of 200 ppm was cytotoxic	Arab et al. (2014)
9.	*Nicotiana tabacum* L.T541	Leaves	AgNPs	96 nm	Biologicalmethod	UV/VIS Spectrophotometer, TEM, XRD and FTIR	10 mg/L	10 mg/L	AgNPs prevent bacterial and fungal infections during explant sterilization	Had potential for removal of microbes. Enhanced viability of protoplasts during isolation	Higher AgNPs concentrations above 10 mg/l caused toxicity	Bansod et al. (2015)
10.	Carnation (*Dianthus caryophyllus* L.)	Nodal segment	AgNPs	------------	Chemical method	UV/VIS Spectrophotometer, TEM, XRD and FTIR	200 mg/L	200 mg/L	Nano-silver exhibits broad antimicrobial activity due to unique physicochemical properties	had no harmful effects on regeneration of explants.	Higher concentrations (300 mg/L) inhibit explant regeneration	Ahmadian et al. (2015)
11.	Stevia (*Stevia rebaudiana Bertoni* cv. Morita II)	Nodal segment	AgNPs	35 ±15 nm	Chemical method	TEM	50 and 100 mg/L	50 and 100 mg/L	AgNPs disrupt fungal cell membranes, impairing infection mechanisms AgNPs inhibit growth and metabolic processes in fungi	Eradication of contaminants during the *in-vitro* culture of plant species	AgNPs may lead to resistance in some fungal strains	Ramírez-Mosqueda et al. (2019)
12.	Barley (*H. vulgare* var. Eunova).	Embryo	AgNPs	------------	------------	------------	6 to 8 mg/L	8 mg/L	Nanosilver interacts at the molecular level in bacterial cells, inhibiting growth	nAg concentrations of 6 and 8 mg/L limit infections and enhance growth Chlorophyll and β-carotene contents increase with nAg addition Higher nAg levels improve mineral content in barley leaves Concentration of 4 mg/L nAg resulted in the longest roots	The addition of 2 mg/L nAg had no effect on infections Limited sourcing of Mn was observed with 2 and 8 mg/L nAg	Krupa-Małkiewicz et al. (2019)
13.	Bare caper or *Karira, (Capparis* decidua FORSK.)	Shoot tips and nodal segments	AgNPs	1.5-15 nm	Biological method (Green synthesis using fruit extract)	UV/VIS Spectrophotometer, TEM and FTIR	150 mg/L	150 mg/L	Nanosilver binds to sulfhydryl groups of enzymes, disrupting metabolic activities	Higher concentrations achieved 100% bacterial and 98.6% fungal decontamination	Immersion in 100 mg/L for 20 or 30 min showed poor survival	Ahlawat et al. (2019)
14.	Date palm (Phoenix dactylifera L.)	Iimmature inflorescences	AgNPs	<70 nm.	------------	------------	0.125-0.5 mg/L	0.5 mg/L	They inhibit ethylene production, enhancing explant survival during micropropagation	0.5 mg/L AgNPs induced the highest callus formation	Contamination persists despite treatments, indicating challenges in tissue culture	El-Kosary et al. (2020)
15.	Olive trees (*Olea* *europaea* L.) cv. ‘Picual’	Nodal segment	AgNPs	6.68-15 nm	Chemical method	UV/VIS Spectrophotometer, TEM	0, 5, 10, and 20 mg /L	5 mg/L	Nano-silver releases tiny silver particles to eliminate microorganisms It inhibits ethylene action, enhancing plant regeneration	Improved the bud sprouting percentage and shoot growth.	Higher AgNPs concentration (20 mg/L) negatively affected growth parameters	Hegazi et al. (2021)
16.	*Aldrovanda vesiculosa*	Shoot tip	AgNPs	----------	-----------	-----------	100-250 mg/L	10 mg/L	Nanoparticles eliminate microorganisms, aiding in disinfection processes	Reduced the contamination rate of explants but noticeably inhibited the regeneration capacity and growth of shoots.	AgNPs led to necrosis of the shoots during regeneration	Parzymies (2021)
17.	Prunus rootstock	Nodal segment	Reduced graphene oxide–copper–silver and silver–selenium	----------	----------	----------	50.00 mg/L Ag and 32.50 mg/L Cu The optimal concentration for AgSe-NPs was 50.00 mg/L Ag and 30.83 mg/L Se	200 mg/L	Antibacterial activity involves metal ion release and direct nanoparticle contact with cells	Concentrations of 100, 200, and 400 mg/L Ag showed 100% inhibition rGO-Cu-Ag and AgSe-NPs effectively reduced *Curtobacterium* sp. at 200 mg/L	Limited antibacterial effect on *Curtobacterium* sp. was noted on the medium surface	Tekielska et al. (2024)
18.	*Ocimum sp.*	Seeds and tissues (leaves, stems and inflorescence)	AgNPs	12-80 nm	Biological method	---------------	10, 50, and 100 mg/L	100 mg/L	Silver nanoparticles disrupt cell membrane structure in microorganisms They lead to depletion of intracellular ATP, causing cell death	Posed no harmful effect on the seeds, tissues and callus induction but rather possess stimulating effect on callus formation at 100 mg/L AgNPs	Lack of optimal sterilization protocols limits tissue culture research samples	Adebomojo and Abdul Rahaman (2020)
19.	Blackberry (*Rubus fruticosus* L.) Navaho variety	Shoot tips	ZnONPs	<35nm	Chemical method	DLS, APS, Zetz potential	10 mg/L	10 mg/L	ZnONPs promote organic acid production, enhancing nutrient absorption	The best growth and development parameters of the plantlets and good biochemical parameters were obtained. Concentrations of 10-30 mg/L ZnONPs increased mineral content	High ZnONP concentrations reduced shoot and root growth in blackberry plantlets Chlorophyll content decreased at 30 and 40 mg/L ZnONPs Increased ZnONPs led to lower phenolic compound content and inhibited morphogenesis and biological quality	Krzepiłko and Matyszczuk (2024)
20.	Olive Cv. Koroneiki, Picual, and Manzanillo	Nodal segment	AgNPs	5-15 nm	Biological method	TEM	5 and 10 mg/L	10 mg/L	Silver nanoparticles bind to cell membrane proteins, causing cell death	High antimicrobial activity, growth promotion, improved stress resistance	-------	Darwesh et al. (2023)
21.	Olive Cv. Koroneiki, Picual, and Manzanillo	Nodal segment	Chitosan nanoparticles	20-50 nm	Chemical method	TEM	40 and 60 mg/L	40mg/L	Chitosan nanoparticles affect cell membrane permeability and inhibit DNA replication	Biodegradability Antimicrobial activity	-------	Darwesh et al. (2023)
22.	Olive Cv. Koroneiki, Picual, and Manzanillo	Nodal segment	Seleniumnanoparticles	15-35 nm	Chemical method	TEM	2.5 and 5 mg/L	2.5 mg/L	Selenium nanoparticles may disrupt protein structure and function, causing oxidative stress	SeNPs show low antimicrobial activity	-------	Darwesh et al, 2023
23.	Gray poplar (*Populus × canescens* Aiton. Sm.)	Axillary bud	AgNPs	70nm	Chemical method	SEM micrograph	0.0015 to 0.003 mg/L	0.003 mg/L	AgNPs exhibit antimicrobial activity	Reduced contamination	Higher concentrations above 15 mg/L are toxic.	Vasyukova et al. (2021)
	Avocado cv. Hass, Fuerte, and Red.	Nodal segment	AgNPs and ZnONPs	--------	----------	------------	40 mg/L	40 mg/l	Silver nanoparticles disrupt cell membrane structure in microorganisms. ZnONPs enhance nutrient absorption	Positively influenced regeneration efficiency, leading to a greater number of shoots, leaves, and increased shoot length per explant	Higher concentration of 80 mg/l is less effective	Shenoda et al. (2024)
24.	Chrysanthemum	*Ex vitro* leaves	AgNPs	< 20 nm	Chemical method	------------	4 mg/L	4 mg/L	AgNP interacts with cellular proteins and DNA, disrupting respiratory functions AgNP induces antioxidant enzyme activities, enhancing plant responses	4 mg/L AgNP achieves 100% medium disinfection	------	Tung et al. (2021)
25.	Mung bean, (*Vigna radiata* L.) specifically Mung NCM-13, MgAT-7, and MgAT-4	Shoot tips, nodal tips and leaf	ZnO NPs and CuO NPs	37.8 nm .	Biological method	UV-Visible spectrophotometer and Particle Size Analyzer	0.5 mg/L	0.5 mg/L	Nanoparticles initiate reactive oxygen species accumulation, activating signaling pathways	Antimicrobial activity and enhanced phytochemical production, indicating a positive effect on regeneration efficiency	------	Iqbal et al. (2022)
26.	*Chrysanthemum × morifolium* (Ramat.) Hemsl., Cvs. ‘UTP Burgundy Gold’ and ‘UTP Pinky Gold’	Shoot tip	ZnO, NPs and ZnO+Ag NPs	25-65 nm, 240 nm and 27-79 nm	Chemical method	XRD, SEM	ZnO+Ag NPs: 400 mg/L	400 mg/L	Nanoparticles alter gene expression, affecting plant physiological processes	Improved the growth parameters of the plantlets	--------	Tymoszuk et al. (2024)

Silver nanoparticles exhibit potent antimicrobial activity against a wide variety of bacteria, fungi and viruses. Their principal mechanisms result from the loss of structural integrity in microbial cell membranes and the production of reactive oxygen species. Other mechanisms of action include the disruption of microbial cell membrane structures, impairment of the electron transport chain, impairment of metabolic pathways for microbial growth, and induction of DNA replication interference, which ultimately leads to cell death (Dakal et al., 2016). These nanoparticles are different from traditional sterilization agents in that they work at low concentrations and do not damage plant tissues (Tung et al., 2021). In several reports, AgNPs have previously achieved high percentages of culture media sterilization and a reduction in microbial contamination (Munkager et al., 2020; Piecková et al., 2021; Wen et al., 2022; Alavi and Ashengroph, 2023).

Nanoparticles such as zinc oxide and titanium dioxide also have antimicrobial activity, which provides more options for sterilization (Abd-elsalam, 2013; Younis et al., 2022). Copper oxide nanoparticle usage also resulted in a 15–25% reduction in the explant infection rate during the initiation phase (Evlakov et al., 2020). Silver nanoparticles had a positive influence on SE and improved plantlet growth, which resulted in improved survival rates under greenhouse conditions (Cuong et al., 2021). There was improved branching of root systems in birch microplants with the introduction of CuO nanoparticles, demonstrating improved adaptive potential. However, some studies have also indicated possible negative effects associated with the introduction of nanoparticles during later stages, such as reduced viability and poor shoot and leaf development (Evlakov et al., 2020). Callus induction is the first step in the regeneration of a plant. This cell calling depends on the availability of important nutrients and growth regulators in the medium. NPs have been shown to increase callus induction *via* increased nutrient assimilation, altered hormonal activity and reduced oxidative stress (Irum et al., 2020; Malik et al., 2021).

Zinc oxide nanoparticles are essential for stimulating callus formation. These nanoparticles enhance cell division and proliferation by providing more macronutrients and micronutrients to the culture medium (Mousavi Kouhi and Lahouti, 2018). These nanoparticles are also treated as signaling molecules that regulate the synthesis of auxins and cytokinins, which are the two major hormones needed for callus initiation (Khan et al., 2022; Lee et al., 2023). ZnO nanoparticles have been demonstrated to improve callus induction in wheat (*Triticum aestivum* L.) by assisting in the uptake of important nutrients and facilitating cell activities that are important for plant growth and development Czyz˙owska and Barbasz, 2019).

NPs also strongly influence the morphogenic capabilities of explants during the initiation stage of micropropagation because of their enhanced growth characteristics, the fate of cell differentiation pathways, and increased pathogen resistance. Their unique properties allow for tailored applications that can optimize *in vitro* conditions for various plant species. In some cases, the presence of specific concentrations of nanoparticles in the nutrient medium, compared with controls, can yield enhanced morphogenic responses, and different plant species have been shown to have a range of optimal nutrient media concentrations to allow enhanced growth (Sichanova et al., 2023).

Gold nanoparticles affect SE by not allowing explant cells to follow a typical differentiation pathway and instead induce the formation of organ-like structures instead of somatic embryos. When the explants are exposed to nanoparticles, the tinkering of their chemical composition of the cell wall occurs, which further affects the developmental pathway (Godel-Je˛drychowska et al., 2023). The advent of nanoparticles into the culture media has decreased the occurrence of infections compared with that of control explants, increasing the survival rates of explants and thereby increasing their adaptability to stressful conditions during the micropropagation process (Evlakov et al., 2020; Pathak et al., 2023). While patterns that emerge from the emergence of nanoparticles may increase morphogenic potential in plants, notable concerns with their level of toxicity and whether nanoparticles have adverse long-term effects on plant health should remain a focus of debate (Pathak et al., 2023).

### 2.2 Shoot proliferation and multiplication stages

The shoot proliferation stage of micropropagation is a crucial phase that occurs simultaneously with shoot formation in cultured explants. The shoot proliferation stage is critical for calculating the overall multiplication rate and productivity of the process. The use of nanoparticles at the shoot proliferation stage of micropropagation has received much research attention, given their potential to facilitate plant growth and development (**Table 2**).

**Table 2:** Effect of nanoparticles on multiplication stage of micropropagation.

S. no.	Plant species	Explant	Type of nanoparticles	Average size of NPs	Synthesis method	Validation and characterization	Optimal concentration/Concentration range	Effective concentration for contamination control	Mechanism of action	Effect on regeneration efficiency	Limitations observed	References
1.	Date palm (*Phoenix dactylifera* L.)	Iimmature inflorescences	AgNPs	<70 nm.	------------	------------	0.125 mg/L	0.5 mg/L	AgNPs influence plant hormones,increasing cytokinins and reducing auxin levels	0.125 mg/L AgNPs showed the highest embryo multiplication and germination	High AgNP concentrations negatively affect globular embryo formation	El-Kosary et al. (2020)
2.	Olive Cv. Koroneiki, Picual, and Manzanillo	Nodal segment	AgNPs	5-15 nm	Biological method	TEM	10 mg/L	10 mg/L	Silver nanoparticles bind to cell membrane proteins, causing cell death	AgNPs positively influence shoot number, length, and multiplication rate	----	Darwesh et al. (2023)
3.	Olive Cv. Koroneiki, Picual, and Manzanillo	Nodal segment	Chitosan nanoparticles	20-50 nm	Chemical method	TEM	60 mg/L	60mg/L	Chitosan nanoparticles affect cell membrane permeability and inhibit DNA replication	Chitosan and nanoparticles showed lesser response for multiplication rate	Higher concentrations of chitosan NPs negatively impacted olive shoot growth	Darwesh et al. (2023)
4.	Olive Cv. Koroneiki, Picual, and Manzanillo	Nodal segment	Selenium nanoparticles	15-35 nm	Chemical method	TEM	5 mg/l	5 mg/l	Selenium nanoparticles may disrupt protein structure and function, causing oxidative stress	Selenium nanoparticles showed lesser response for shoot multiplication rate	Selenium NPs showed detrimental effects on several olive cultivars' shoot growth	Darwesh et al. (2023)
5.	Gray poplar (*Populus × canescens* Aiton. Sm.)	Axillary bud	AgNPs	70nm	Chemical method	SEM micrograph	0.0015 to 0.003 mg/L	0.0015 to 0.003 mg/L	AgNPs exhibit antimicrobial activity against phytopathogens in tissue cultures	Reduced contamination and accelerated shoot growth	----	Vasyukova et al. (2021)
6.	Banana (*Musa acuminata* L.) (cultivar Grand Nain)	Shoot tip	AgNPs	80 nm to 100 nm	------------	SEM	12 mg/L	12 mg/L	AgNPs induce oxidative stress through ROS generation	Three fold increase in shoot growth parameters	Higher concentrations above 12 mg/L are inhibitory	Tamimi and Othman (2023)
7.	*Nardostachys jatamansi*	Shoot part	AuNPs	11.1±1.9 nm for citrate-AuNPs and 19.5±3.2 nm for CTAB-AuNPs	Chemical method	TEM	For citrate-AuNPs: 60 µM and for CTAB-AuNPs: 40 µM	40 µM	AuNPs improve antioxidant activities and biomass production Differentially charged AuNPs affect gene expression related to growth and defence	Modulation of gene expression, enhanced antioxidant activity	Positively charged AuNPs enhance micropropagation more than negatively charged AuNPs	Joshi and Joshi (2024)
8.	*Chrysanthemum × morifolium* (Ramat.) Hemsl., Cvs. ‘UTP Burgundy Gold’ and ‘UTP Pinky Gold’	Shoot tip	ZnO and ZnO+Ag NPs	25-65 nm, 240 nm and 27-79 nm	Chemical method	XRD, SEM	For ZnO+Ag NPs: 400 mg/L	400 mg/L	Nanoparticles may modify cellular membranes and macromolecules They influence cell division and defense systems	Improved the growth parameters of the plantlets, including shoot fresh.	--------	Tymoszuk et al. (2024)
9.	Sweet Basil (*Ocimum basilicum* L.)	Leaves	Cu-NPs	20-40 nm	Chemical method	UV/VIS spectrophotometry and TEM	5 µM	5 µM	----------	Enhanced somatic embryogenesis	Cu-NPs above 5 µM showed reduced effectiveness	Ibrahim et al. (2019)
10.	*Stevia rebaudiana* Bert.	Nodal segment	Silver Nanofibers (Ag-NFs)		Chemical method		1 to 50 mg/L	1 to 50 mg/L	Reduce oxidative stress by neutralizing free radicals . They influence antioxidant enzyme activity in plants	NF1-Ag salt increased shoot height and biomass accumulation significantly	Higher concentrations (100 mg/L) reduced growth parameters	Sichanova et al. (2023)

Silver nanoparticles have been shown to increase the mean number of fresh shoots per explant and the number of explants that produce shoots due to an ethylene blocking mechanism (Aghdaei et al., 2012). Copper oxide and silver nanoparticles reduce the infection rate of explants and increase their morphogenic ability after explants are added to the culture medium (Evlakov et al., 2020). Potassium nanoparticles (K-NPs) working at optimal concentrations significantly increase the number of microtubers formed in potato cultivars (Farrag et al., 2024).

Nanoparticles, which primarily include gold (Au), silicon (Si), and carbon-based nanomaterials, have been shown to significantly increase shoot proliferation because of their ability to increase phytohormone levels and increase nutrient transport (Kim et al., 2017; Talebi, 2018). Gold nanoparticles have been shown to stimulate the activity of cytokinin, a hormone that facilitates shoot development. Research has shown that when these nanoparticles are introduced into a culture medium, the quantity and length of shoots increase significantly (Jadczak et al., 2019; Joshi et al., 2022). In addition, silicon nanoparticles can reduce oxidative stress by instigating antioxidant enzymes, which can help promote an environment for shoot proliferation (Mukarram et al., 2022). Carbon-based nanoparticles, such as graphene oxide (GO) and carbon nanotubes (CNTs), have shown potential as agents of stimulation for shoots. These factors are known to improve water and nutrient absorption, cell wall elasticity, and the transport of growth regulators, thus allowing for increased shoot multiplication (Mathew et al., 2021).

High doses of nanoparticles may inhibit plant viability and growth, such as in shoots and leaves, which limits the number of conversions (Evlakov et al., 2020). Additionally, a high dose of nanoparticles could stimulate auxin-influenced branching and root development despite negatively affecting health (Tymoszuk et al., 2024).

NPs can enhance shoot proliferation and overall plant quality; however, it is necessary to recognize that the effects of NPs are concentration-specific and that their overuse can lead to detrimental side effects. This duality emphasizes that optimization is necessary in micropropagation protocols. However, even after initial optimization, there are potential nanotoxicity risks and biosafety concerns associated with the use of nanoparticles in agricultural applications (Pathak et al., 2023).

### 2.3 Rooting stage

Rooting is another significant step in micropropagation. An established root system leads to good plantlets. This phase often requires auxin-rich media; however, roots can be formed more efficiently via nanoparticles (Talebi, 2018). The authors of studies on various species have shown that nanoparticles can greatly increase plant growth and rooting efficiency during the rooting stage of micropropagation (**Table 3**). The use of silver nanoparticles at the optimal concentration (12 mg/L), significantly increased both root count and length during banana micropropagation experiments (Tamimi and Othman, 2023). In strawberry micropropagation, the use of AgNPs decreased the level of ethylene accumulation, suggesting an improvement in plantlet quality that would facilitate root development and improve survival (Tung et al., 2021). Silver nanoparticles additionally improved the disinfection of strawberry explants along with the reduction of pathogen infestation and disease, and therefore, importantly, contributed to the establishment of a healthy and robust root system for woody species such as gray poplar (Vasyukova et al., 2021). Zinc oxide nanoparticles in combination with auxins greatly increased rooting percentages in seedling apple microcuttings and resulted in increased root length and decreased callus formation (Alizadeh and Dumanoglu, 2022). Iron oxide nanoparticles, silicon nanoparticles and graphene oxide have all joined the ranks of previously mentioned nanoparticles that have been widely studied for root growth promotion (Triphati et al., 2021; Zhao et al., 2022), whereas iron oxide nanoparticles are particularly influential on root induction and improve nutrient availability, auxin perception and signaling, and activate antioxidant defense pathways leading to increased root formation (Rai et al., 2022). Silicon nanoparticles have a complementary function in root development. They improve phosphorus and potassium assimilation, two nutrients that are directly related to root development, and improve root tissue structural integrity (El-Kady et al., 2017). More recently, silicon nanoparticles have been shown to protect from abiotic stress related to root formation. These NPs have been shown to effectively increase the growth and biomass of strawberry roots (Sener et al., 2023). Graphene oxide is a carbon-based nanomaterial that may promote rooting by increasing the ability of plants to take up water and transport nutrients. It interacts with auxins, facilitating cell elongation and division and allowing rooting for many different types of plant species (Cheng et al., 2016; Zhao et al., 2022).

**Table 3:** Effect of nanoparticles on rooting stage of micropropagation.

S. no.	Plant species	Explant	Type of nanoparticles	Average size of NPs	Synthesis method	Validation and characterization	Optimal concentration range	Effective concentration for contamination control	Mechanism of action	Effect on regeneration efficiency	Limitations observed	References
1.	*Arabidopsis thaliana*	Seed	AgNPs	-----------	Chemical method	--------------	40-80 mg/L	80 mg/L	Silver ions bind to thiol groups, causing protein denaturation	Improved plant growth and the reduction of fungal and bacterial contamination	Concentrations above 100 mg/L showed undesirable effects on plant growth	Soltanloo et al. (2010)
2.	Gray poplar (*Populus × canescens* Aiton. Sm.)	Axillary bud	AgNPs	70nm	Chemical method	SEM micrograph	0.0015 to 0.003 mg/L	0.0015 to 0.003 mg/L	---------	Reduced contamination and also stimulated root system formation	With an increase in the concentration of AgNPs, a tendency towards a decrease in the rate of rhizogenesis was observed	Vasyukova et al. (2021)
3.	Banana (*Musa acuminata* L.) (cultivar Grand Nain)	Shoot tip	AgNPs	80 nm to 100 nm	------------	SEM	12 mg/L	12 mg/L	AgNPs induce oxidative stress through ROS generation	Three fold increase in root numbers and root length	Higher concentrations above 12 mg/L are inhibitory	Tamimi and Othman (2023)
4.	*Nardostachys jatamansi*	Shoot part	AuNPs	11.1±1.9 nm for citrate-AuNPs and 19.5±3.2 nm for CTAB-AuNPs	Chemical method	TEM	The optimal concentration for citrate-AuNPs is 60 mM and for CTAB-AuNPs is 40 mM	40 µM	AuNPs improve antioxidant activities and biomass production Differentially charged AuNPs affect gene expression related to growth and defence	CTAB-AuNPs provide better oxidative stress resistance under climatic changes Gene ontology analysis shows CTAB-AuNPs affect more biological processes	Positively charged AuNPs enhance micropropagation more than negatively charged AuNPs	Joshi and Joshi (2024)
5.	Apple (*Malus domestica* Borkh.) cultivar #67,	Microcuttings	ZnO NPs and ZnO NPs loaded with Auxins (IAA-nZnO and IBA-nZnO)	<50 nm	Chemical method	FTIR-ATR, Zeta potential, SEM, EDX, TEM and DTA.	1.0 mg/L IBA-nZnO	1.0 mg/L	Nanoparticles release active substances in a controlled manner and their physicochemical properties influence plant rooting success	IBA-nZnO significantly improved rooting percentages	Rooting failure in apple genotype #67 with nZnO at low concentrations	Alizadeh and Dumanoglu (2022)

Even though nanoparticles provide favorable advantages for micropropagation, it is important to assess the potential for toxicity and the environmental concerns associated with the use of materials at the molecular level. However, once again, given the potential outcomes of improved rooting, we need to consider any ecological concerns when utilizing this new form of plant biotechnology in the form of nanotechnology.

### 2.4 Acclimatization

The last micropropagation step is acclimatization, in which the plantlets are cultured *in vitro* and then moved to natural conditions. Acclimatization is a step where there is a risk to the plantlets for mortality due to desiccation, pathogen infection, and stressful environmental conditions. The acclimatization process, where a plant survives, can be improved with nanoparticles by increasing stress tolerance and antimicrobial protection from pathogens (El-Saadony et al., 2022). The use of nanoparticles influences the acclimatization and hardening steps in micropropagation (**Table 4**). The inclusion of AgNPs has been shown to improve the plant growth rate, increase the survival rate, and decrease microbial contamination, facilitating the transition from *in vitro* to *ex vitro*. These nanoparticles have been shown to improve somatic embryo formation and the overall number of somatic embryos in *Panax vietnamensis* (Cuong et al., 2021).

**Table 4:** Effect of nanoparticles on hardening/acclimatization stage of micropropagation.

S. no.	Plant species	Explant	Type of nanoparticles	Average size of NPs	Synthesis method	Validation and characterization	Optimal concentration range	Effective concentration for contamination control	Mechanism of action	Effect on regeneration efficiency	Limitations observed	References
1.	Gray poplar (*Populus × canescens* Aiton. Sm.)	Axillary bud	AgNPs	70nm	Chemical method	SEM micrograph	0.003 mg/L	0.003 mg/L	Preventing rotting of the root system under the influence of pathogenic microflora.	Increased the survival rate of microclones by 45%.	------	Vasyukova et al. (2021)
2.	*Hemidesmus indicus* (L.) R. Br. ex Schult.	Nodal segment	SiNPs	------------	Chemical method	-------------	1.0 mg/L	1.0 mg/L	SiNPs improve chlorophyll synthesis and photosynthesis rates	SiNPs induced positive morpho-anatomical changes in plantlets This study is the first to report SiNPs' quantitative and qualitative benefits	Higher concentrations (2.0 mg/L) hinder growth	Manokari et al. (2024)
3.	Potato (*Solanum tuberosum* L.) cv. Spunta	Sprouts	CS-NPs	36.58 nm	Chemical method	DLS, XRD and TEM	100 mg/L	100 mg/L	Regeneration response increased with (CS-NPs) concentration up to 250 mg/L. Histopathological changes confirmed (CS-NPs) effectiveness against PVY in potato shoots	Nanoparticles influence plant response based on composition and size	Higher concentrations (250-300 mg/L) caused phytotoxic effects	Elsahhar et al. (2022)

For blueberry micropropagation, the addition of AgNPs improved shoot propagation rates, which suggests that there is potential for improving growth during acclimatization (Tejada-Alvarado et al., 2022). Compared with those of the control plants (44.44%), the survival of the plants exposed to silver nanoparticles was much greater (93.65%) (Cuong et al., 2021). AgNPs are functional sterilants that minimize microbial contamination of growth media, which is crucial for successful acclimatization. The use of these nanoparticles in chrysanthemum micropropagation resulted in better acclimatization under greenhouse conditions, leading to earlier developmental stages (Tung et al., 2021). More intensely, AgNPs act to protect plants during this acclimatization process from fungi and bacterial pathogens. These nanoparticles serve to limit microbial infections and diminish each plant’s risk of loss following transplantation (Gautam et al., 2020; Tariq et al., 2020).

In general, silicon nanoparticles enhance acclimatization by improving water use efficiency, the strength of plant cell wall composition and photosynthetic efficiency, allowing plants to acclimatize better post-transplantation to environmental stresses (Moraes and Lacava, 2022). The advantages of the use of nanoparticles in micropropagation are remarkable however, some studies have noted that excessive amounts of nanoparticles can also have negative effects on plant morphology and development therefore, the precise fine-tuning of nanoparticles throughout various acclimatization processes could be highly important (Evlakov et al., 2020).

## 3 Nanoparticles and their action mechanisms in micropropagation

**Figure 2:**
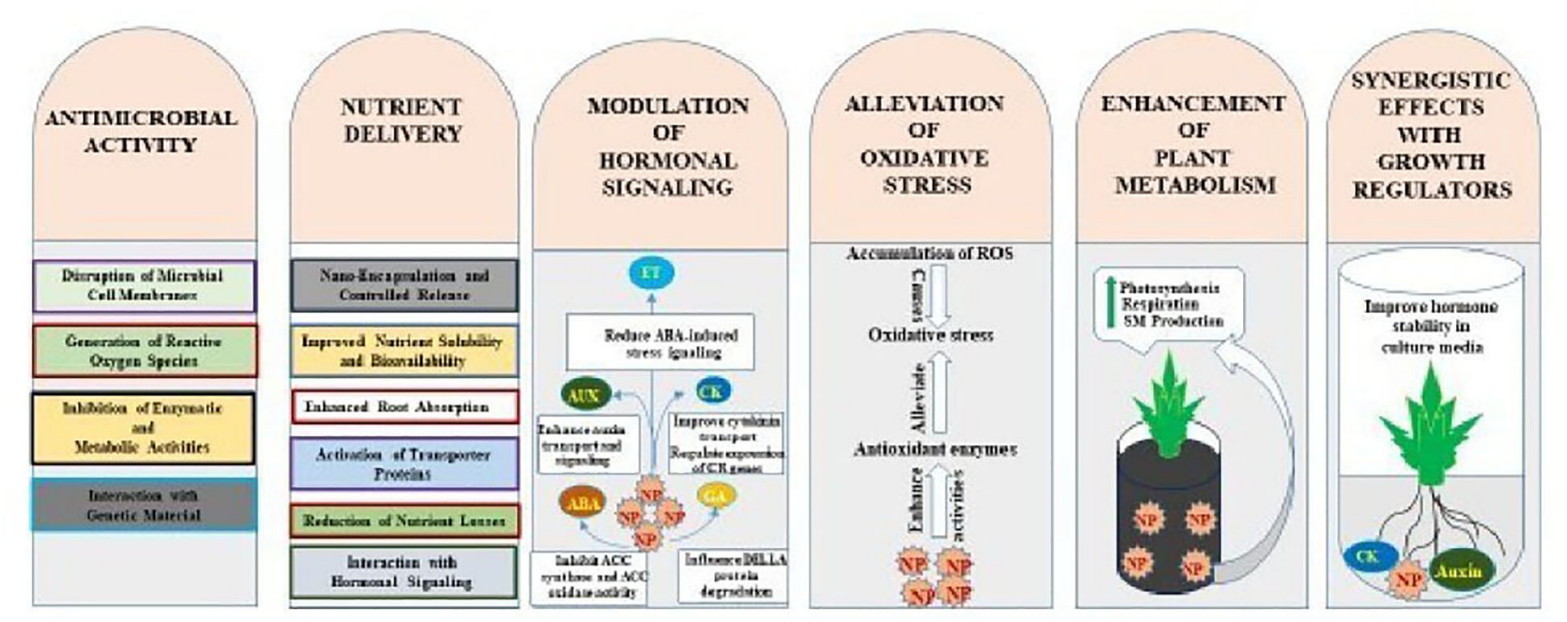
Action mechanisms of nanoparticles in micropropagantion.

**Figure 3:**
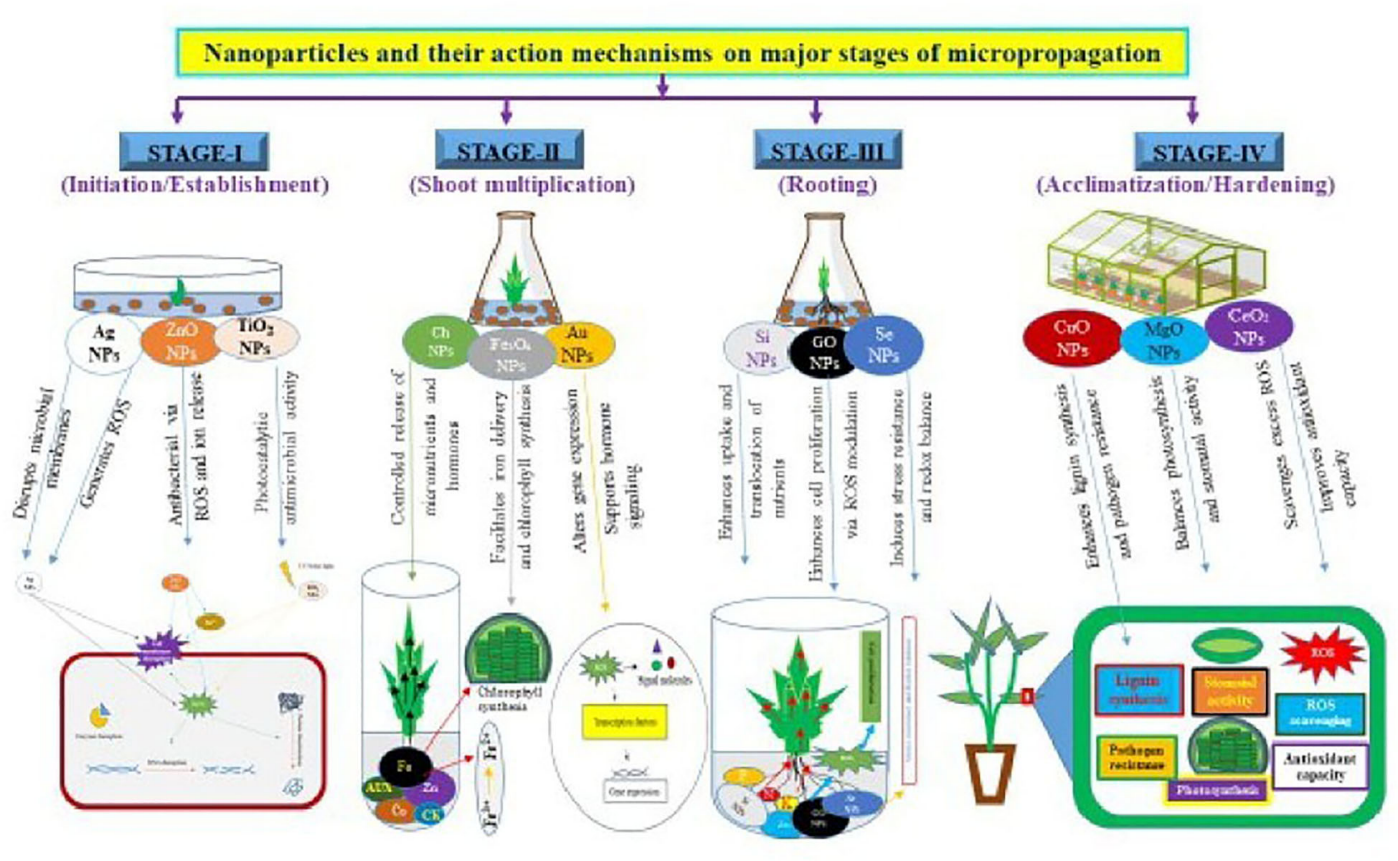
Nanoparticles and their action mechanisms on major stages of micropropaganation.

Nanoparticles have emerged as promising tools for enhancing micropropagation efficiency. In plant tissue culture, their integration addresses persistent challenges such as microbial contamination, nutrient delivery, oxidative stress, and suboptimal morphogenesis (Kim et al., 2017). The mechanistic actions of nanoparticles in micropropagation can generally be classified into five primary functional areas (**Figure 2**): (i) antimicrobial activity, (ii) enhancement of nutrient delivery, (iii) modulation of hormonal signaling, (iv) alleviation of oxidative stress, and (v) activation of metabolic pathways. These mechanisms are extensive and overlap while cumulatively improving propagation efficiency, contamination resistance, acclimatization probability, and optimal growth (Mostafa et al., 2018; Rawat et al., 2018). The action mechanisms of important nanoparticles in major stages of micropropagation are shown in **Figure 3**.

### 3.1 Antimicrobial activity

Microbial contamination, mostly by fungi and bacteria, is a continued barrier in plant tissue culture and results in large financial losses in the sector, while also ending with somatic origins that would later become genetically regulated plants. Traditional chemical disinfectants are normally effective, but they can cause phytotoxicity as well as residual toxicity. Hence, the use of inorganic nanoparticles with antimicrobial properties has presented itself as a superior alternative to chemical sterility agents. Because of their multidimensional characteristics, metals and metal oxides (AgNPs, ZnO NPs, CuO NPs, and TiO_2_ NPs) have inherently potent broad-spectrum antimicrobial activity *via* different mechanisms (Ranjan and Ramalingam, 2016; Kim et al., 2017; Fatima et al., 2021; Rahmawati et al., 2022; Kalra et al., 2022). Metallic nanoparticles, particularly silver and copper nanoparticles, have some effective antimicrobial properties that may reduce disease incidence by 50% (Francis et al., 2024).

The fundamental antimicrobial action of these nanoparticles is based on ROS (e.g., superoxide anions (O_2_^-^), hydroxyl radicals (•OH) and hydrogen peroxide (H_2_O_2_)). ROS can disturb microbial membranes, denature cellular proteins, and interfere with nucleic acids, leading to cell death (Kaur et al., 2023). Although silver nanoparticles are mostly composed of elemental silver (Ag), their surfaces may be functionalized with chemicals, which may also shed light on antimicrobial mechanisms. The interaction of AgNPs with thiol groups and phosphorus-containing compounds can lead to conformational changes and functional inactivity of these structural molecules. Importantly, these nanoparticles also permeabilize bacterial membranes, which also triggers cytoplasmic leakage and apoptosis (Bondarenko et al., 2018).

Both ZnO and TiO_2_ NPs have photocatalytic properties related to the induction of light-excited electrons into holes that can catalyze ROS. The antimicrobial capabilities of all types of nanoparticles, including ZnO and TiO_2_, are driven by their size at the nanoscale, surface area, and ability to interact intimately with microbial cells. The impressive capability of these phytocompatible drug nanotherapies for use in media sterilization means a stronger reliance on such methods than harsh disinfectants characterized by high toxicity (Zakharova et al., 2019).

### 3.2 Nutrient delivery

Tissue culture media contain a balanced mix of macronutrients and micronutrients; however, the nutrient bioavailability is often still limited by precipitation processes, pH swings, and chemical interactions. Nanoparticles offer a novel approach to nutrient delivery, with potential advantages in terms of greater solubility and bioavailability (Panpatte et al., 2016; Jampilek and Králová, 2017; Tariq et al., Singh et al., 2024c). Novel engineered nanofertilizers can entrap essential nutrients or chelate them and deliver them directly to plant cells, bypassing barriers and enhancing uptake efficiency (Gohar et al.; Ghazaryan et al., 2024, simultaneously improving nutrient uptake in both the apoplastic and symplastic pathways (Khlebnikova et al., 2023; Djanaguiraman et al., 2024).

In *in vitro* propagation, iron oxide nanoparticles (Fe_34_ NPs) have been successfully utilized to alleviate iron deficiency. Iron oxide nanoparticles can deliver Fe³^+^ ions in a controlled and sustained manner. This is important since Fe is critical for the biosynthesis of chlorophyll, electron transport, and a myriad of enzymatic functions (Feng et al., 2022). In addition, silicon nanoparticles positively contribute to the mechanical strength of cell walls, fortify cell walls, and increase stress tolerance, particularly under drought conditions, through regulating water retention and enzyme activities (Dhakte et al., 2022).

Researchers have also recently reported nanocarriers for macronutrients such as phosphorus (P) or potassium (K), which may be useful because of their limited ability in soil. Phosphorus in hydroxyapatite or other phosphate-bound nanocarriers assists with ATP synthesis and the metabolism of nucleic acids. Potassium-containing nanoparticles have been reported to regulate osmotic potential and activate enzymes. Overall, these advances improved nutrient uptake, promoting enhanced development of shoots and roots through various aspects of micropropagation (Ditta and Arshad, 2016).

### 3.3 Modulation of hormonal signaling pathways

Throughout micropropagation, phytohormones function to coordinate cellular differentiation, organogenesis, and plant morphogenesis. NPs alter phytohormone interactions by modifying biosynthesis, transport, sensitivity, and hormone-associated signaling pathways. Hormonal signaling modulation by NPs can take place either by direct contact with the hormone itself or indirectly through the transcriptional regulation of hormone-responsive genes.

In auxin (indole-3-acetic acid, IAA) signaling, which is critical for root induction, the addition of Fe_34_ NPs and graphene oxide NPs increases the level of auxin signaling itself. In both cases, it not only enables the polar transport of auxins but also increases the expression of auxin efflux carrier genes to improve meristematic activity and root organogenesis (Cheng et al., 2016; Yan et al., 2020, Yan et al., 2020; Yadav et al., 2022). As another example of an increase in hormone response and activity, AuNPs were found to have a synergistic effect on cytokinin, promote shoot proliferation, translocate cytokinins by stabilizing, and increase multiplication and nodal development *via* signals from cytokinins. The combination of nanoparticles and hormones in the micropropagation of plants is encouraging, especially because of the interaction with fewer hormones, where some nanoparticles can exert hormone-like effects, while some can work synergistically with exogenous hormone applications and generally have the prospect of better adjustment of hormone balance without the risk of adding more chemical burden to the media (Vinkovic et al., 2017).

Notably, NPs interact directly with abscisic acid (ABA) and gibberellins (GA) and can regulate responses to stress as well as elongation growth (Quesada, 2022). Other nanoparticles may even mimic hormonal action alone or be additive to exogenous hormone treatments and provide new methods to manipulate hormonal balance without increasing the chemical load (Kandhol et al., 2022).

### 3.4 Alleviation of oxidative stress

During the micropropagation process, oxidative stress commonly occurs due to high levels of ROS. The sources of oxidative stress include wounding, the application of exogenous hormones, and the artificial environmental components of *in vitro* cultivation (Hancock et al., 2001). In addition to acting as signaling molecules at lower concentrations, ROS can denature proteins, lipids, and nucleic acids at higher concentrations, leading to potentially nonviable plant tissue (Bhattacharjee, 2019). NPs can elicit these two types of ROS. They can produce ROS (especially at very high doses), but for the most part, they can increase the level of oxidative stress, which increases the activity of antioxidant defense systems (Kumar et al., 2018; Sachdev and Ahmad, 2021).

Silicon nanoparticles increase SOD, CAT, and POD enzyme activity to reduce oxidative damage and restore redox homeostasis (for example, during shoot elongation and root induction phases) (Mukarram et al., 2022). ZnO NPs and TiO_2_ NPs also effectively scavenge ROS, thereby protecting tissue as viable and reducing pentane (Singh and Chaudhary, 2020; Faizan et al., 2021).

When applied to stress-responsive genes, nanoparticles have been shown to provide upstream signaling for salicylic acid, jasmonate (JA), and ethylene for establishing systemic plant protection. This elicitor-like activity not only enhances the poor defense of plants but also further promotes their survival in stressful plant tissue cultures (Khlebnikova et al., 2023; Francis et al., 2024; Djanaguiraman et al., 2024).

### 3.5 Enhancements in plant metabolism and secondary metabolite production

Plant metabolism includes three common processes: photosynthesis, respiration and secondary metabolism; together, these processes serve as the basis for biomass accumulation, as well as adaptive responses to the environment. NPs affect these processes at the molecular and physiological levels, although the stage of the *in vitro* to ex vitro transition (i.e., the acclimation/hardening phase) is critical (Selvakesavan et al., 2023; Sharma and Chauhan, 2023). The application of Si NPs, TiO_2_ NPs and CeO_2_ NPs has been shown to improve photosynthetic efficiency, enhance chlorophyll contents and improve nutrient use efficiency (Gohar et al., 2024).

Photosynthetic systems are affected by TiO_2_ nanoparticles, as they increase photocatalytic activity in chloroplasts, promoting improved light absorption and better coupling to electron transport, which leads to an improved photosynthetic rate and increased stomatal conductance (Didaran et al., 2023). Similarly, Si NPs play seminal roles as osmotic buffers and induce structural changes in cell walls; these roles enhance stress resistance and ‘water retention’. These effects improve the overall ‘vigor’ and capacity for uniform hardening and acclimation of the plant (Siddiqui et al., 2020).

Furthermore, nanoparticles act as elicitors of secondary metabolite pathways, stimulating the production of phenolics, flavonoids, alkaloids and terpenoids, which are important for various facets of plant defenses and medicinal activities in plants (Sreelekshmi et al., 2022; Savanur, 2022; Inam et al., 2023).

While the use of nanoparticles as enhancers for micropropagation offers increased capacity to bioenhance multiple factors, including plant growth, re-establishment and rooting into natural growth media, it highlights the necessity for thorough attention to practical use in terms of nanoparticle dose-response relationships, phytotoxicity, and ecological cascading effects. Continued expansion of knowledge on plant–nanoparticle interactions are integral for understanding the molecular-level mechanisms that improve plant growth systems (Sharma et al., 2022; Khlebnikova et al., 2023).

## 4 Methodological framework with nanoparticle application

As the use of nanotechnology within the field of plant micropropagation continues to increase, the ability to improve propagation efficiency, disease resistance, nutrient transport, and overall plant performance continues to emerge. However, to ensure reproducibility, safety, and efficacy, an explicit methodological framework regarding the application of nanoparticles in plant tissue culture is needed. More specifically, the framework should include standardized characterization methods, methods of determining dose–responses, safety assessment techniques, and quality assessment protocols that minimize variability in outcomes, toxicity risks, and potential regulatory challenges when adopting/ascribing to nanobiotechnology in agricultural biotechnology and commercial plant propagation as a whole (Cinelli et al., 2016; Oomen et al., 2018; Mohamed and Kumar, 2019).

### 4.1 Standardization of nanomaterial characterization

An integral component of this framework is the standardization of nanomaterial characterization. Characterization ensures that the different physicochemical properties of NPs, which impact their biological performance, are relatively well defined and consistently reproduced. The key characterization parameters include the particle size distribution, morphology, surface area, zeta potential, chemical composition, and surface functionalization. Tools commonly employed for NP characterization include transmission electron microscopy (TEM), scanning electron microscopy (SEM), dynamic light scattering (DLS), X-ray diffraction (XRD), Fourier transform infrared spectroscopy (FTIR) and the Brunauer–Emmett–Teller (BET) surface area (Ramachandran et al., 2021).

A standardized characterization protocol should begin by measuring the particle size and distribution, which influences cellular uptake and reactivity. While DLS is a common technique because of its ability to analyze dispersed particles, it should always be followed up with TEM or SEM to obtain morphological confirmation. The next important measure is the zeta potential. The zeta potential helps characterize the coalescence of nanoparticles and their possible interactions with the plant cell membrane, providing further characterization of the nanoparticles. A relatively large absolute zeta potential (typically above ± 30 mV) reflects good coalescent particle stability, which is essential for determining the consistency of bioavailability in suspension culture media (Varenne et al., 2015a). The chemical composition and crystallinity of a nanoparticle is also an important part of its characterization because these parameters determine the rate of dissolution, the ion release profile and the redox potential of metal-based nanoparticles. These parameters may be characterized *via* XRD and energy-dispersive X-ray spectroscopy (EDX) (Reed-Gore et al., 2018; Deepty et al., 2019). The surface functional groups present on the nanoparticles matter because they contribute to the overall biocompatibility and plant hormone-mimicking capability of the nanoparticles to influence cellular signaling. The surface chemistry must also be characterized, which can be accomplished *via* FTIR or X-ray photoelectron spectroscopy (XPS). All these parameters must be reported by the researchers in detail. These guidelines must be followed when publishing and are recommended by the OECD Working Party on Manufactured Nanomaterials (WPMN), or ISO/TC 229 (Rasmussen et al., 2016).

### 4.2 Dose-response determination techniques

In addition to valuable characterization, dose–response determination approaches are integral to understanding the concentration-dependent responses exerted by NPs during micropropagation. Low dosages of NPs such as silver, zinc oxide, or graphene oxide may promote callus induction, root formation, or shoot elongation, but high dosages often react to oxidative stress, genotoxicity, and loss of growth. As a means to define practical application limits, reliable replications of dose-response curves must be developed. Thus, a tiered experimental design needs to be developed that includes several concentrations of the NPs (0.1 to 100 mg/L), multiple time points (24 h to weeks), and separation of the culture stages (initiation, proliferation, rooting, and acclimatization). The biological endpoints should consist of quantitative parameters such as callus mass, shoot number, root length, chlorophyll content, and antioxidant enzyme activities (SOD, CAT, POD) and stress markers (MDA, H_2_O_2_ levels). Additionally, transcriptomic and proteomic profiling can provide mechanistic details related to the dose-dependent modulation of signaling and metabolic pathways (Claeys et al., 2014; Kumar et al., 2016).

Aquiring clearance projections from the start, as well as acute and chronic exposure models, for both acute and chronic dose-response analyses. For example, some acute responses may only be seen as immediate toxicity, whereas chronic low doses could very easily lead to discrete epigenetic or physiological responses that occur cumulatively over successive subcultures. Advanced statistical models such as nonlinear regression, NOAEL (No Observed Adverse Effect Level) estimates, and benchmark dose (BMD) analysis can provide insight into safe and non-harmful nanoparticle concentrations, particularly when combined with *in vitro–in vivo* correlation studies, where plantlets initially treated *in vitro* are subsequently followed following acclimatization, and further bolster the ecological relevance of dose to indications (Nikinmaa, 2014; Savage et al, 2019).

### 4.3 Safety assessment protocols

Safety assessment protocols represent another critical element of the framework. It is much broader, as it demands attention beyond phytotoxicity to consider shifts in the microbial community, the expression of genes, and long-term ecology. For plant tissue culture, safety assessment should ultimately begin with cytotoxicity tests using a cell viability assay, such as 2,3,5-triphenyl tetrazolium chloride (TTC) or MTT, with suspension cultures or calli (Karakas et al., 2017). Genotoxicity tests, including the comet assay, micronucleus assay, and RAPD-PCR, may reveal DNA damage due to exposure to nanoparticles and, as such, can serve as a guide for the risk of chromosomal instability or unintended changes that can occur under somaclonal variation (Verdon et al., 2022). In addition, oxidative stress assays, which are used to determine the levels of ROS and lipid peroxidation, and assays of antioxidant enzymes produce a biochemical safety profile (Shukla et al., 2012; Gašparović et al., 2013; Agarwal et al., 2014).

At the microbial level, the unforeseen actions of various nanoparticles could also affect positive endophytes or rhizosphere consortia, either in the process or after hardening. Thus, culture-dependent and culture-independent techniques (e.g., qPCR and 16S rRNA sequencing) are useful for assessing microbial diversity in treated and control samples (Chhipa, 2023). Transgenerational studies, although infrequently published, are equally important for determining latent or heritable effects of NP exposure on seed germination, plant vigor, and reproductive outcomes. Future toxicology assessments will benefit from these multigenerational safety data to obtain regulatory clearance and general acceptance (Poma et al., 2014).

### 4.4 Quality control measures

QC measures must be taken during the entire process of NP production and applied to end-use studies. QC starts with the NP synthesis stage and must ensure consistency between different batches, and along a line, the particle size, charge and composition must be ensured. For green-synthesized NPs, extract variability from plants is a barrier to consistency; hence, standardization of the source material is needed (e.g., at the minimum age of the plant, the plant part used, or the solvent system). However, for all NPs, the sterility, endotoxin (most importantly for bioderived NPs) and stability must be assessed, including storage assessments. Where possible, certificates of analysis (CoAs) should be reported listing, at a minimum, all critical physicochemical properties (Maity et al., 2021; Saleh and Fadillah, 2023).

During application in micropropagation systems, media preparation must follow sterile, reproducible conditions, with uniform NP dispersion achieved *via* sonication or mechanical stirring. To avoid aggregation or precipitation, NP suspensions should be freshly prepared or stabilized *via* biocompatible agents (e.g., citrate and polyethylene glycol). The risk of cross-contamination between treatment groups should be minimized through spatial or temporal separation and rigorous tool sterilization. Regular checks for NP leaching, sedimentation, and degradation in culture media are essential to ensure consistent bioavailability (Kim et al., 2017; Gupta et al., 2020b).

Internal QC audits should involve routine calibration of instruments (e.g., pH meters, laminar flow cabinets), validation of assay protocols (e.g., controls in antioxidant enzyme tests), and replication of experiments across different labs or technicians (Kinns et al., 2013; Zhuravleva, 2014). Documentation standards, including detailed lab notebooks, digital records, and standardized reporting templates, support transparency and traceability. Furthermore, collaboration with accredited nanotechnology or toxicology laboratories for independent verification of findings adds robustness to the dataset and aids in regulatory compliance (Elberskirch et al., 2022).

As the field matures, there is a growing call for harmonized guidelines and databases for nanoparticle use in plant biotechnology. This includes establishing repositories for NP physicochemical and biological data, similar to the eNanoMapper or OECD NanoStat database. Such initiatives can help in the predictive modeling of NP behavior, reduce redundancy, and promote responsible innovation. The methodological framework described here aims to lay a foundation for such harmonization, ensuring that nanotechnology in plant tissue culture evolves along safe, effective, and scientifically rigorous lines (Kokina and Plaksenkova, 2022; Naidoo, 2022).

## 5 Standardization and protocol development

The reproducibility of nanoparticle assimilation into plant micropropagation systems is based on the elaboration of well-defined standardized protocols in terms of synthesis and characterization, use, and follow-up (Ankamwar et al., 2020). Standardization and protocol are key for the future of the translation of nanobiotechnology, not only to ensure scientific rigour but also to obtain regulatory acceptance, industrial scalability and environmental safety (Shen and Wang, 2011; Haydon, 2015) (Varenne et al., 2015b; Roubert et al., 2016). Without standardized protocols, fragmented results plague industry, resulting in nonreproducible results and inconsistent biological responses, which hinders both academic advancement and commercial opportunity (Boslough, 2013; Weller, 2021).

The intricate behavior of NPs, which is often influenced by variables such as size, shape, surface charge, propensity for aggregation, chemical composition, and their interaction with the biological matrix, requires detailed, stepwise, and context-specific standardization regimes. Such frameworks  need to span disciplines, accommodate interspecies variation in plant response to 81 stresses, and ensure comparability between laboratory and field conditions (Ensor, 2011). Standardization must be performed via nanoparticle synthesis itself, at which point batch-to-batch reproducibility is still at the interface of what is feasible (Mülhopt et al., 2018). NPs may be produced *via* physical, chemical or biological (green) methods, and the resulting particles  display different physicochemical characteristics (Triphati and Pirzadah, 2023).

Chemical synthesis methods usually produce well-defined particles; however, they can employ toxic precursors and hazardous solvents, whereas green synthesis using biological materials, such as plant extracts or microbial metabolites, is more ecofriendly but less controllable (Sharma and Sharma, 2021). The creation of standard syntheses involves the exact determination of precursor concentrations, reaction times, pH and temperature conditions and purification steps. These parameters together have to be tuned and synchronized to generate nanoparticles with a uniform size, size distribution, morphology, and surface functionalization. Otherwise, small differences during synthesis can have a profound effect on the reactivity, stability, and bioavailability of NPs, resulting in vastly different physiological effects when applied to tissue culture. Thus, it is essential to adhere to good manufacturing practice (GMP) principles for the production of nanoparticles for use in agriculture (Hühn et al., 2017; Sarlak and Abdi, 2022).

In the follow-up to particle synthesis, thorough characterization of the particles is essential and should be an integral aspect of the standardized procedure. Physicochemical characterization is essential for verifying the desired properties of nanoparticles and enables the correct interpretation of the biological effects of the system. Standard parameters that must be described include particle size (determined by dynamic light scattering or transmission electron microscopy), zeta potential (indicative of surface charge and colloidal stability), crystalline structure (e.g., by X-ray diffraction), functional groups (e.g., by means of FTIR spectroscopy) and surface area (by BET analysis) (Hodoroaba et al., 2020).

Furthermore, dispersibility in water and plant tissue culture media should be checked via UV–vis spectroscopy and dynamic light scattering. These properties determine the biological behavior of NPs, including their uptake efficiency by explants, their translocation through plant tissues or their interaction with components of media, such as sucrose or phytohormones (Moore et al., 2015; Rani et al., 2022). Characterization should be performed both before and after incubation in culture media to be aware of possible aggregation, dissolution or chemical modification. Without this, experimental interpretations are speculative, and dose-response relationships cannot be definitively quantified. Consequently, maintaining scientific transparency and reproducibility in any protocol of nanoparticle application in micropropagation, including characterization as a mandatory module, is crucial (Boverhof and David, 2010).

Following synthesis and characterization, the subsequent hurdle is developing protocols for the application of nanoparticles for the various stages of micropropagation. In developing protocols, standardization should consider plant species, genotype, developmental stage, and environmental conditions. Silver nanoparticles can again be employed to sterilize a culture vessel, which normally involves immersion for 5–10 min to ameliorate microbial contamination in explants (Hien et al., 2021), whereas zinc oxide nanoparticles can be added directly into the culture media to stimulate root elongation or mitigate oxidative stress (Molnár et al., 2020; Singh et al., 2022, Singh et al., 2023). Specific application methods (immersion, incorporation into culture media, and aerosols) and the frequency of application must be carefully defined, and exposure concentrations should be optimized with dose-response assays. Protocols should also contain control treatments that are subject to the same conditions as the treatment but lack the application of the nanoparticles to attribute any biological responses only to the nanoparticles. Another area of standardization is developing thresholds for usable and safe concentrations of nanoparticles. As a result, nanoparticles can produce biphasic (hormetic) effects by being beneficial at lower doses and toxic at higher doses; thus, cytotoxicity assays, oxidative stress markers such as malondialdehyde content or antioxidant enzyme activities, and viability assays such as TTC completely define the range of effective concentrations. For example, these concentration intervals should consider the plant species being tested, as well as the specific tissue type (e.g., leaf, node, meristem), since the biological responses of plants can vary tremendously (Tirumala et al., 2021).

Standardizing the composition of media containing nanoparticles is also important. Tissue culture media are complex mixtures of different sugars, salts, vitamins, hormones and gelling agents. These components can also react with nanoparticles, sometimes causing aggregation, chemical changes, or perhaps unintended bioactivity. Thus, standard protocols should indicate the compatibility of NPs with media components *via* stability studies that consider time, temperature and light exposure. The protocols should also describe how the media were prepared, including the order of addition of the NPs (before and/or after autoclaving), pH adjustments, and how mixing was performed, to avoid nanoparticle precipitation and/or degradation. Another consideration is NP-hormone interactions since nanoparticles could have synergistic or antagonistic effects with endogenous or exogenous plant growth regulators (PGRs), such as auxins and cytokinins. The standard protocols should also incorporate factorial experimental designs to evaluate the realized PGR and NP concentrations to optimize various morphogenetic responses, such as shoot proliferation or the promotion of a root system (Moore et al., 2015; Alagoz et al., 2022).

An important, although sometimes overlooked, consideration for standardization is quality control and reproducibility across laboratories. Validations of protocols in advance of publication should be institutionalized as inter-laboratory validations, comparing a common nanoparticle formulation and micropropagation protocol in different geographic or institutional settings. Ring trials in this way would allow for the identification of protocol-sensitive parameters and provide the framework for universally applicable SOPs. There is also a need for harmonization around documentation and reporting standards. Researchers should be expected to provide complete metadata, including but not limited to the source of the nanoparticles, route of synthesis, batch number, storage conditions, media composition, and biological data. Centralized databases or repositories of NP characterization and bioactivity in micropropagation would also facilitate meta-analyses and contributions to evidence synthesis efforts. They could also advance predictive modeling initiatives, such as machine learning efforts, to establish recommended nanoparticle types and concentrations for a given plant species and culture conditions (Roubert et al., 2016).

At the same time, there must be a robust biosafety element integrated into protocol development. Each standardized protocol will leave room for evaluations of both safety to the environment and safety to humans, typically ranging from ecotoxicity to the accumulation of residuals in regenerated plants. The proposed protocols will include long-term studies on the stability of nanoparticle-treated plantlets, for example, their soil stability (if returned to the environment) and leaching of NPs after being placed in the environment. These studies will have to be part of the protocol life cycles, as institutions will have to assess compliance with regulations but also the acceptance of nanotechnology by non-expert audiences and scientists alike, who need access to standardized protocols to study and regulatory experimental evidence as potential lifesaving technologies. By including safety endpoints such as assessment of DNA integrity, photosynthetic efficiency, and microbial community characterization in acclimatized plantlets, standardized NP protocols would have a greater degree of acceptability and robustness (Shende and Takke, 2021; Sharma et al., 2022).

Ultimately, protocol development has the possible future of creating integrated modular protocol packages such as diagnostic kits or vaccine platforms that are made of pre-validated modules (synthesis, characterization, application, safety, and data reporting modules) and could be tailored to the crop type and micropropagation phase. These kits are sellable by biotechnology companies and serve nurseries, agricultural research stations, and tissue culture laboratories. These kits increase the standardization of NP protocols, decrease the learning curve for non-expert researchers, and facilitate nanotoxic plant propagation protocols in agriculture (Mishra et al., 2022). Moreover, widely accepted protocols systematized by an internationally recognized body (e.g., FAO, ISO, BIS, and ICAR) with common characteristics will allow for community trust in the methods, along with assisting in the creation of policy and streamlining regulations (Bas et al., 2021).

## 6 Challenges and risks to nanoparticles in micropropagation

The use of nanoparticles in micropropagation has received considerable attention because of their unique physicochemical characteristics and ability to improve tissue culture results. Despite their advantages, there are numerous problems associated with implementing NPs in plant biotechnology systems. Given the nanoscale size and high level of reactivity of NPs, they can enable the precise delivery of nutrients, hormones, and antimicrobial agents within plant growth media. Conversely, nanoscales may also have unintended consequences for plant physiology and environmental and human health. This section reviews the key concerns raised when applying nanoparticles in micropropagation, including uncertainties in dose–response, environmental fate, regulatory deficiencies, economic limitations and standardization.

### 6.1 Toxicity thresholds and dose-response relationships

The biological efficacy and safety of nanoparticles in micropropagation are related to their dose-related response, which constitutes one of the greatest challenges in nanobiotechnology. NPs possess certain unique properties depending on their application in micropropagation, as they have a much higher surface area-to-volume ratio, are reactive and contain properties based on quantum mechanics, unlike traditional agrochemicals, which alter their association with plant tissues. These characteristics can promote or prevent the micropropagation process, as they have the potential to encourage growth in plants or raise concerns regarding phytotoxicity (Ramkumar et al., 2022). Whereas low concentrations of some NPs, such as AgNPs, ZnO NPs and Fe_34_ NPs, can stimulate growth or differentiation processes in the form of shoot proliferation, rooting and somatic embryogenesis, all of the above NPs select for cellular damage after certain thresholds involving oxidative stress, membrane leakage and genotoxicity (Irum et al., 2020; Tymoszuk et al., 2022).

A major complication in the analysis of dose-response relationships is related to the plant species and their developmental stages and the physical and chemical properties of the nanoparticles (Rawat et al., 2021). The size, shape, surface charge, and functionalization of nanoparticles can change their bioavailability and uptake (Ma et al., 2013). Smaller nanoparticles are more likely to simply traverse cell membranes and quickly be sequestered in organelles, whereas larger nanoparticles can end-up outside of the cell, with the potential to interfere with metabolic and signaling pathways (Mosquera et al., 2018). However, nanomaterials can generate ROS concentrations that are high-enough to potentially lead to oxidative stress, which may have a deleterious effect on cell viability, leading to lipid peroxidation, protein denaturation, and DNA damage (Manke et al., 2013).

The absence of a therapeutic index for the use of nanoparticles in plant systems has encouraged their use in micropropagation protocols. An index of the therapeutic window for dosage in pharmaceutical therapies has been established, but we do not have a similar range of dosages that are considered safe and efficacious from nanoparticles in plant systems (Kumari et al., 2022). Moreover, the interaction of nanoparticles with plant growth regulators can further complicate inference regarding optimal dosages. Therefore, specific treatments in a systematic manner for any one or a range of species are needed to assess potential concentrations that are intended to maximize efficacy with minimal cytotoxic effects. Longer-term studies may also be beneficial for identifying whether repeat doses through subcultures, or across generations, result in the collection of toxic effects or stress-related adaptations of the plantlets (Kandhol et al., 2022).

The inconsistency in experimental designs reported in the literature, including everything from the composition of culture media utilized to the environments imposed during our experiments, adds another layer of complexity to the establishment of dose-response curves (Cvrčková et al., 2015). Without standardized protocols, cross comparison of the impact of nanoparticles is an unreliable process. We need to standardize bioassay protocols that have measurable endpoints (i.e., chlorophyll concentration, root/shoot biomass, electrolyte leakage and enzyme activity). Advances in molecular diagnostics and imaging, such as transcriptomics and proteomics, could reveal valuable information on the sublethal effects of nanoparticles, which could help appropriately define toxicity thresholds and limits of exposure (Kang et al., 2023).

### 6.2 Environmental fate and bioaccumulation

The environmental impacts of the use of nanoparticles in plant micropropagation are becoming more significant, particularly with respect to their persistence, mobility and bioavailability through bioaccumulation in ecosystems. If nanoparticles are added to plantlets in the *in vitro* phase, once they are assembled into the *ex vitro* environment, they can be transferred to soils or aquatic environments, including the direct transfer of plantlets, disposal of culture media, or leaching from *ex vitro* additions. After being released into the environment, nanoparticles may undergo physical/chemical changes, such as aggregation, dissolution, biomolecular and macromolecular association and involvement, all of which have a compound effect on their fate and ecological toxicity (Batley et al., 2013; Wojcieszek and Ruzik, 2022).

Bioaccumulation in trophic chains may be one of the most serious hazards. For example, silver, copper, and zinc oxide nanoparticles, among others, may persist in soil matrices, become associated with, and be taken up by, plant roots and microbial communities and subsequently translocated into edible flora/plant tissues, thus entering the food chain. Numerous studies have shown the bioavailability of engineered nanoparticles with agricultural crops, which raises the possibility of engineered nanoparticles accumulating in higher organisms, potentially including humans. Many of the studies have focused primarily on those crops grown in soils, but the same hazards may also exist for crops derived from tissue cultures after acclimatization and following transplantation despite having undergone, in addition, planting medium recognition/realization (Murali et al., 2022).

The ecotoxicological effects of nanoparticles are not limited to only plants but also to soil microorganisms, fungi, nematodes, and aquatic organisms (Chhipa, 2021). For example, silver nanoparticles are used as *in vitro* antimicrobial agents but can inhibit nitrogen-fixing bacteria and interfere with the mycorrhizal relationships necessary for plant uptake of nutrients (Cao et al., 2017; Yu et al., 2021) in addition to causing unplanned collateral damage. These effects may negatively impact soil fertility and crop yield over time. Furthermore, nanoparticles that enter water can have a negative impact on the aquatic environment. Once in aquatic systems, they reduce photosynthetic productivity in algae, promote bioaccumulation in fish, and impair the coral cycle in invertebrates.

Methodologically, environmental monitoring of nanoparticles is an additive challenge because of their nanoscale size; they are uniquely variable in chemical composition and often interact with disparate environmental matrices. The environmental risk assessment tools are designed primarily for bulk chemicals. When attempting to add the exposure and effects of nanomaterials, this approach is not straightforward, and the use of complex compounds is unlikely to yield the same results as those of bulk chemical assessment methods. We need more advances in detection methodologies, such as isotopically labeled nanoparticles or synchrotron spectroscopy electronics, single particle inductively coupled plasma mass spectrometry (spICP-MS), and other more sophisticated methodologies to track the compartmental distribution and quantify the accumulation of nanoparticles within biological tissues and environmental compartments (Cornelis et al., 2021; Singh et al., 2024d).

There are at least two main methods for addressing environmental risks related to the use of nanoparticles in micropropagation. First, synthesis methods that are deemed more environmentally friendly and that use plant-based or microbial principles to make biodegradable or less persistent nanoparticles should be used whenever possible. Second, pre-acclimatization procedures should be able to degrade or eliminate any nanoparticles from plant tissues prior to field transplantation. Government regulatory agencies need to have some type of framework for the disposal of nanoparticles (more research related to this topic is needed). This is particularly important for laboratories and greenhouses where researchers use nanoparticles, and there are risks of unintended release into the environment (Kim et al., 2017).

### 6.3 Regulatory landscape and approval pathways

Governmental efforts to establish regulatory frameworks for the secure, uniform and ethical practice of nanoparticles in agriculture and biotechnology cannot seem to keep up with the acceptance of nanoparticles in micropropagation systems. The specific physicochemical traits that nanoparticles exhibit create complexities for regulatory frameworks that have relied on typical bulk assessment. Nanoparticles behave differently than typical agrochemicals because their size and reactivity are dependent, and the different interactions of nanoparticles depend on their performance, provider and biological system, so they do not assess nanoparticles in the same manner as traditional agrochemicals do. This gap in regulations has created a significant bottleneck to scaling up nanotechnology with plant tissue culture (Kumari et al., 2023).

Most regulatory authorities have not developed eligible and scientifically validated regulations specific to the use of nanoparticles in plant biotechnology. The few regulations that do exist, such as the EU REACH (Registration, Evaluation, Authorization and Restriction of Chemicals) regulations or U.S. EPA documents that discuss the usage of this nanomaterial, focus on its industrial usage rather than its applications in agricultural biotechnology. Consequently, researchers and commercial end-users practice in a regulated or informal regulated state that creates inconsistencies in applications, safety and quality control, including labeling. Furthermore, there are exposed liability concerns for institutions that use nanoparticle-based micropropagation methods without completed risk assessments (Ngarize et al., 2013; Bajpai et al., 2020).

The lack of standardized testing protocols only adds to the regulatory issue. Even if laboratories follow unique procedures to produce, characterize, and test nanoparticles, they compile different and heterogeneous datasets that are not representative, reliable or comparable, or aggregable in meta-analysis. The heterogeneity and endpoint variation make it much more difficult for regulators to determine the maximum acceptable exposure or the bioequivalence of different formulations of nanoparticles. Although international organizations responsible for developing protocols, such as the International Organization for Standardization (ISO) or the Organization for Economic Cooperation and Development (OECD), have developed high-throughput screening protocols for characterizing and testing nanoparticles as they relate to toxicology, many in plant sciences are just emerging (Gomes et al., 2021; Bleeker et al., 2023).

Risk assessment pathways for nanoparticles used in micropropagation should also include assessments of acute and chronic toxicity, in conjunction with ecological information, and the consequences of any genotoxicity and lifecycle assessment data. If regulatory pathways are developed to assess the safety of nanoparticles, they must also allow for any interactions with and how nanoparticles will function as plant growth regulators or other components of the culture media, as well as any potential synergies or antagonistic consequences. We need to be able to determine the direct or indirect effects (or absence) of nanoparticles on gene expression and epigenetic stability in regenerated plants, particularly when regenerated plants are consumed as food (Ranjan et al., 2021).

Tackling these issues involves active and cross-disciplinary collaboration. Researchers, regulators, and industry will have to combine their efforts to produce a comprehensive regulatory regime that will cater to the unique properties of nanoparticles. This needs to include premarket approval processes, required testing for safety, post-market surveillance of use and market disclosure requirements. An expanded role for regulatory agencies will be in producing guidelines for required documentation on nanoparticle-based culture media and formulations; labeling, disclosure and transparency within the value chain; and traceability.

### 6.4 Economic feasibility and scalability analysis

The economic viability and scale of nanoparticles for use in micropropagation are two of the most important but still underexplored dimensions for understanding why nanoparticles have not gained wider use and acceptance in commercial horticulture and plant biotechnologies (Wu and Li, 2022). The documented benefits of the use of nanoparticles, such as increased shoot multiplication, reduced contamination, increased rooting efficiency and stress tolerance, are well known under laboratory conditions (>500 papers), but determining how to obtain practical laboratory benefits or advantages for economically viable large-scale operations is challenging. This is primarily due, in part, to the costs incurred from producing, stabilizing, characterizing and integrating nanoparticles into tissue culture procedures (Abdelmajeed and Aboul-Nasr, 2013).

NPs are generally produced through energy-hungry physical or chemical processes that are generally costly due to expensive precursors, reducing agents and specialized instrumentation (ultrasonication, high-temperature furnaces, autoclaves, and reactors are just a few examples). While greener synthesis approaches using biological templates (such as plant extracts or microbes) are emerging, they have not achieved the consistency or scale of manufacture to be widely adopted in industrial applications. The functionalization of nanoparticles with appropriate ligands is fundamental to creating nanoparticles with stability, specificity, and controlled release, which also adds to production costs. In general, tissue culture laboratories (especially when they operate in low-resource situations or with non-commercial or low-margin crops such as trees) are unlikely to ever recoup the financial costs of adopting a consistently reliable nanoparticle-based protocol, regardless of any direct benefits such as production efficiency, improved quality (e.g., improved propagation accuracy), throughput or other secondary benefits from the normalization of nanoparticle use (Afonso et al., 2024).

When undertaking cost analyses, one must always consider how nanoparticle systems will fit with the current infrastructure. Most tissue culture laboratories have no existing experience with nanoparticle dispersion, characterization, or tracking, and the introduction of nanoparticles as a component in media preparation, sterilization and delivery will most likely require new methods, new equipment, new personnel training, and even significant changes to how waste is managed (e.g., disposal of residues of nanoparticles used in media preparation), so the associated costs to the operating budget, while likely not relevant when capital costs are considered, must be justified by justifiable measurable returns, such as propagation efficiencies, product quality (e.g., improved accuracy of propagation), throughput, or additional value for normalizing the use of nanoparticles (Tariq et al., 2020; Su et al., 2022).

In addition, the viability of using nanoparticles in micropropagation *via* economies of scale is also not understood. While many small-scale studies demonstrate the efficacy of a few milligrams or micrograms of nanoparticles, the process of scaling up to kilograms for commercial propagation, with millions of plantlets, creates many challenges with sourcing nanoparticles in the same form, preventing agglomeration of the nanoparticles, and obtaining batch consistency. These challenges could ultimately result in inconsistent responses of the nanoparticles and negatively impact product quality, as well as the trust of stakeholders and end-users alike (Kokina and Plaksenkova, 2022).

The cost-benefit ratio is also related to the type of crop being produced. For high-value crops, including orchids, elite fruit cultivars, and medicinal plants, any increase in propagation efficiency or improved plant resistance to disease increases the costs associated with nanoparticles. Low-margin crops, including leafy vegetables and fodder plants, may not be able to provide sustainable economic viability for practices that rely on costly nanoparticle protocols. Crop economic modeling provides a precise number that will equate break-even and profitability under different crop production scenarios (Zaman and Maitra, 2018).

Things that might help reduce economic feasibility include reusable nanoparticle matrices, time-released formulations to reduce waste, or the use of nanocomposites added to culture vessels or media gels to minimize the need for repeated applications. The use of various precision delivery systems, such as microfluidics, nanofiber scaffolds or encapsulated growth matrices, may lead to a gain in the delivery efficiency of these nanoparticles, indicating that the delivery of fewer nanoparticles will result in a problematic dose. Relationships with nanomaterial suppliers and some investments in local manufacturing will increase the cost of nanoparticle usage and access (Delgado, 2009).

With respect to some costs, if they are deemed in the best interest of governments or funding bodies, regulation, partnerships with nanotechnology regarding public and private relations, and international collaboration might also increase the likelihood of making lower cost barriers for adoption and the transfer of technologies. As an example, government and funding bodies explicitly subsidizing green nanoparticle synthesis as examples being used for agricultural uses or adding nanotechnology to the scopes of innovation-related schemes in agricultural use signifies alternatives to support the engagement of facilitating technologies for utilization. Generally, a multidisciplinary approach involving nanotechnology, economics, plant science, and industrial engineering means that knowledge of nanoparticle-assisted micropropagation should grow to be a meaningful, scalable, and economically useful technology in common culture.

### 6.5 Standardization challenges in synthesis and application

Importantly, standardization is a key component of scientific reproducibility and industrial scalability. Without standardization, we would be unable to scale up the production of nanoparticles or their integration into micropropagation. The inconsistencies in nanoparticle synthesis, characterization, and, ultimately, their application in horticulture are the primary impediments to integrating nanoparticles into the micropropagation process because they make it impossible to compare and establish best practices across studies. Furthermore, such inconsistencies mean that regulatory bodies are unable to regulate these substances or assess their risk and prevent the development of effective, crop specific, nanoparticle protocols (Zhang and Ge, 2018).

The range of nanoparticles used in plant tissue culture can vary significantly on the basis of core material (e.g., silver, gold, zinc oxide, iron oxide, silicon), morphology (e.g., spherical, rod, flower-like), size distributions, surface charges, and levels of functionalization. Small differences in synthesis conditions (e.g., pH, temperature, reactant concentration, reducing agents) can lead to large variations in the properties of nanoparticles and, consequently, in their biological effects. Small nanoparticles may penetrate cell walls more easily but could be more phytotoxic. However, without standardized protocols, researchers cannot reproduce research findings or transfer technology commercially (Mishra et al., 2022).

In addition to problems with synthesis variability, there is an absence of standard protocols for the characterization of nanoparticles. Techniques such as DLS, TEM, SEM, zeta potential measurements and UV-Vis spectroscopy have been used with varying degrees of application by researchers, including differing application parameters and reporting methods. These various methods lead to variable conclusions on the size, stability, and reactivity of nanoparticles, which makes it difficult to predict their biological outcomes. Most published studies report nominal concentrations (mg/L), without evaluation or reporting on the actual bioavailable fraction of nanoparticles in culture media. This would be informative in establishing their functional relevance (McNeil, 2011).

The application methods are also variable. Plant cultures can be exposed to nanoparticles in solid or liquid culture media *via* foliar spraying, seed priming, or coatings on the surfaces of culture vessels. The form of exposure, timing, time spent, frequency, etc., are also generally variable among the studies, leaving little to no common strategy for these variables across the literature. This inconsistency limits the generalizability of the results and complicates efforts to optimize nanoparticle use for specific micropropagation stages, such as callus induction, organogenesis, rooting, or acclimatization (Wohlmuth et al., 2022).

When these issues are considered, a priority is to create consensus standards regarding how particles are synthesized, characterized, and utilized in micropropagation. Creating a standard operating procedure (SOP) will require explicit descriptions of the physicochemical characteristics of the particles, protocols for the dispersion of particles, sterilization protocols and storage conditions. There is also potential for academic institutions, industry, and regulatory authorities in collaboration to develop certified reference materials (CRMs) and validated test methods related to agri-nanotechnology.

An additional important aspect of standardization includes incorporating nanotoxicology assessments into standard operating procedures. Considerations for standardized assays for cytotoxicity, genotoxicity and ecotoxicity should be included in the routine evaluation of nanoparticle formulations and could include high-throughput screening approaches and new omics-based technologies in search of elicited sub-lethal/long-lasting effects on plant physiological processes and genetic integrity for nanoparticles used in micropropagation (Koedrith et al., 2021; Pokhrel et al., 2021).

There should also be efforts toward digital standardization, and centralized databases or even metadata repositories where researchers can deposit and retrieve nanoparticle characterization, exposure conditions, and biological responses would be beneficial in considerably enabling meta-analyses, machine learning-based predictive modeling, and the evolution of evidence-based protocols (Elberskirch et al., 2022).

## 7 Future prospects: overcoming current barriers

Despite several challenges, the future of nanoparticles in micropropagation appears promising, especially with ongoing developments, innovations and interdisciplinary integration.

Emerging eco-synthesis methods, such as green synthesis using biological agents, are being developed as viable green alternatives to more conventional chemical approaches. These alternative syntheses are cost-effective and reduce hazardous byproducts while providing greater long-term environmental benefits. Functionalization approaches allow for the modification of the surface of nanoparticles, increasing their targeting efficacy, bioavailability and interaction with plant tissues, all of which may allow for the delivery of “smart” nanoparticles for the controlled release of nutrients, plant growth regulators, or antimicrobial agents while further improving efficient micropropagation outcomes.

The possibility of integrating nanoparticles with imaging methods (fluorescence microscopy or Raman spectroscopy) to study interactions in plant tissue in real-time is extremely exciting. It will be important to use these tools to deliver and implement nanoparticles properly, as they are related to performance. In addition, the combination of nanoparticles with genetic engineering tools such as CRISPR-Cas systems provides the opportunity to deliver genes, alter traits, and develop disease resistance. Additionally, nanoparticle-enabled sensors could enable precision agriculture, as we can start to monitor micropropagation environments to coordinate and optimize growth conditions, increase efficiencies, and reduce human error.

Nanotoxicity also needs to be reported in future research, as it is related to the biodegradability, mobility, and long-term impacts of nanoparticles in plant systems and ecosystems. It will be important to have biocompatible and degradable nanoparticles that do not bioaccumulate or persist. Multiple generations of studies need to be conducted to assess chronic exposure risk, especially with food crops. There is a need for nanotechnology researchers to be cautious with ecologists and plant biotechnologists to develop a safety profile for the use of nanomaterials in agriculture.

The cost associated with the production of nanoparticles decreases as production methods improve, and there is increasing industrial interest in biological-based processes. Recent developments, such as large-scale bioreactors and green synthesis methods, can facilitate the mass production of nanoparticles while reducing their impact on the environment. The more uniform and disease-free plant materials that are needed, particularly for high-value crops, there will continue to be commercially viable methods to utilize nanoparticles for micropropagation.

Nanoparticles are being investigated for new uses beyond conventional micropropagation, including somatic embryogenesis, cryopreservation and gene delivery. In some of these applications, particularly cryopreservation, nanoparticles can provide further cryoprotective functions *via* structural stabilization of the cell during freezing or enhance the formation of somatic embryos by enhancing the differentiation of the cells. Applications that use nanoparticles with these new applications offer tremendous possibilities and future perspectives for plant breeders and tissue culturists.

Nanomaterials have offered tools to increase nutrient uptake, increase disease resistance and thus overall performance, which decreases the dependency on synthetic agrochemicals. In addition, when used in micropropagation, they can be seen as sustainable agricultural practices since they improve propagation rates while reducing contamination and improving plantlet quality while providing a lower environmental footprint. With declining land availability and increasing food demands, nanomaterials or nanoparticles in conventional propagation schemes may contribute to resilient, resource-efficient crop production systems.

## 8 Conclusion

The incorporation of nanoparticles in micropropagation systems represents a breakthrough in plant tissue culture, providing new potential solutions for many traditional and existing stresses, including microbial contamination, nutrient restriction, hormonal imbalance, and environmental sensitivity during acclimatization. The characteristics of NPs, including their size, surface reactivity, and diverse physicochemical profiles, have the potential to improve nutrient uptake, increase phytohormone signaling capacity, alleviate oxidative stress, and increase shoot and root biomass proliferation, which contributes to the efficiency of plant regeneration. Nevertheless, several scientific, environmental, and regulatory questions remain. We still face many issues, not the least of which are the lack of optimal protocols for use, variability in species response to plant NPs, the potential for cytotoxicity at higher doses in micropropagation, and ecological issues such as bioaccumulation. Therefore, there is a need for a multidisciplinary systems approach to plant biology that combines nanotechnology, plant biology, toxicology, and environmental sciences to develop safe, scalable, and sustainable NP micropropagation systems for global food production. Future research should develop NP green synthesis, clearly defined NP assessment, and standardized regulatory frameworks. If proper due diligence between NP systems, global food production, and sustainable agriculture is attained, NPs can potentially transform plant biotechnology and provide real assets for sustainable agriculture, horticulture, and food security.
